# Supplementary material for: Protocols of Thiamine Supplementation: Clinically Driven Rationality vs. Biological Commonsense
Source: J Clin Med. 2025 May 28;14(11):3787. doi: 10.3390/jcm14113787 (PMC12156702; doi:10.3390/jcm14113787)
Supplement: Supplementary file 1 [file jcm-14-03787-s001.zip › Supplementary file S2.pdf]

Protocols of thiamine supplementation: clinically driven rationality vs biological commonsense. Supplementary data

Calculations:

Daily dose = IV(Dose x Times_daily x Duration) + PO(Dose x Times_daily x Duration)
Daily dose (corrected) = IV(Dose x Times_daily x Duration) + PO(4.5mg x Times_daily x Duration)

Total dose = sum of Daily doses
Total dose (corrected) = sum of Daily doses (corrected)

Daily dose (x-fold) = Daily dose (corrected) / Whole-body store
Total dose (x-fold) = Total dose (corrected) / Whole-body store
with whole-body store, range 20 to 30 mg

Mean daily dose = Daily dose / Duration of treatment (IV+PO)

Example:
Protocol recommending: 250mg IV thrice a day for 3 days, then 250mg PO per day for 3 days
Daily dose = 250x3x3+250x1x3 = 1800 + 750 = 2550mg per day
Daily dose (corrected) = 250x3x3+4.5x1x3 = 1813.5 mg
